# Conditional expression of human β-hexosaminidase in the neurons of Sandhoff disease rescues mice from neurodegeneration but not neuroinflammation

**DOI:** 10.1186/1742-2094-9-186

**Published:** 2012-08-04

**Authors:** Stephanos Kyrkanides, Sabine M Brouxhon, Ross H Tallents, Jen-nie H Miller, John A Olschowka, M Kerry O’Banion

**Affiliations:** 1 Department of Children’s Dentistry, Stony Brook University, Stony Brook, NY, 11894-8701, USA; 2 Health Science Center, Stony Brook University, Stony Brook, NY, 11794-8701, USA; 3 Department of Dentistry, University of Rochester School of Medicine & Dentistry, Rochester, NY, 14642, USA; 4 Neurobiology & Anatomy, University of Rochester School of Medicine & Dentistry, Rochester, NY, 14642, USA

**Keywords:** GM_2_ gangliosidosis, β-hexosaminidase, Mouse, Neuron, Sandhoff disease, Transgenic

## Abstract

This study evaluated whether GM_2_ ganglioside storage is necessary for neurodegeneration and neuroinflammation by performing β-hexosaminidase rescue experiments in neurons of HexB^−/−^ mice. We developed a novel mouse model, whereby the expression of the human HEXB gene was targeted to neurons of HexB^−/−^ mice by the Thy1 promoter. Despite β-hexosaminidase restoration in neurons was sufficient in rescuing HexB^−/−^ mice from GM_2_ neuronal storage and neurodegeneration, brain inflammation persisted, including the presence of large numbers of reactive microglia/macrophages due to persisting GM_2_ presence in this cell type. In conclusion, our results suggest that neuroinflammation is not sufficient to elicit neurodegeneration as long as neuronal function is restored.

## Introduction

The pathognomonic feature of Sandhoff disease is GM_2_ ganglioside storage primarily in neurons. Catabolism of GM_2_ ganglioside in mammalian cells is undertaken by β-hexosaminidase, a lysosomal acidic hydrolase. Structurally, β-hexosaminidase (HEX) is comprised of two subunits, α and β, and exists in three isoforms HEXA (α/β heterodimer), HEXB (β/β homodimer) and HEXS (α/α homodimer). In humans, HEXA (α/β) catabolizes GM_2_ presented by a third protein named GM_2_ activator. Each subunit, α and β, is encoded by a distinct gene, *HEXA* and *HEXB*, located on human chromosomes 15 and 5, respectively. Human patients with *HEXA* (Tay-Sachs) or *HEXB* (Sandhoff) mutations develop storage of GM_2_ ganglioside in the lysosomes due to the lack of HEXA (α/β) enzyme activity [1]. Although HEXA enzyme is present in all cell types and tissues, neurons are characterized by remarkably higher concentrations of gangliosides than other cell types and therefore are highly susceptible to GM_2_ lysosomal storage. Similarly to humans, two murine genes encoding for β-hexosaminidase have been identified: *HexA* and *HexB*[1]. Targeted deletion of the *HexA* locus resulted in the development of a mouse phenotype that showed only a mild degree of the expected pathology and lack of any neurological findings [1]. In contrast, disruption of the murine *HexB* locus resulted in a mouse phenotype that closely resembled that of the human disease. The mice displayed storage of GM_2_ ganglioside in the central nervous system (CNS), and neurons with membranous cytoplasmic bodies similar to those in Tay-Sachs and Sandhoff patients [1,2]. The phenotypic variation between humans and mice appears to result from differences in the ganglioside degradation pathway between the species. It has been proposed that a second ganglioside degradation pathway exists in the mouse, whereby GM_2_ can be, at least in the absence of HEXA (α/β), metabolized by a murine sialidase to asialo-GM2 and subsequently catabolized by HEXB (β/β). However, human sialidases cannot metabolize GM_2_ ganglioside. Therefore, in the mouse HexB disruption results in GM_2_ gangliosidosis, whereas in the human either HEXA or HEXB mutations can cause GM_2_ storage. For these reasons, the HexB^−/−^ knockout mouse is widely accepted as the appropriate animal model in the study of GM_2_ gangliosidosis [1,2].

Clinically, HexB^−/−^ knockout mice display, similarly to human patients, a near-normal phenotype at birth, but quickly develop muscle weakness, rigidity, and motor deterioration typically leading to death at approximately 4 months of age [1,2]. Progressive neuronal GM_2_ storage is a cardinal characteristic of the HexB^−/−^ mice, followed by neurodegeneration associated with neuronal apoptosis occurring in the central nervous system [3]. Additional studies have demonstrated the presence of reactive microglia and astrocytes in the HexB^−/−^ brain, along with increased levels of inflammation-related genes [4-7]. Deletion of macrophage-inflammatory protein (MIP)1α retarded neurodegeneration in the HexB^−/−^ mouse model [8]. Moreover, transplantation of healthy bone marrow to HexB^−/−^ pups attenuated microglia activation, reduced the extent of neuronal apoptosis and ameliorated the clinical phenotype [5,9]. As the presence of activated microglia preceded neuronal cell death and reactive microglia were observed proximal to neurons undergoing apoptosis, it was hypothesized that neuroinflammation contributes to neurodegeneration [4,8]. Neuroinflammation and reactive microglia have also been associated with neurodegeneration in several other neural diseases [10,11]. Taken together, the aforementioned studies suggest that microglia activation and neuroinflammation are contributory to neurodegeneration.

Recent studies in our laboratory focused on the role of infiltrating peripheral blood mononuclear cells (PBMC) in the HexB^−/−^ brain. We demonstrated that inhibition of PBMC infiltration in the brain after deletion of the C-C chemokine receptor type 2 (CCR2) protein resulted in partial reduction of neuroinflammation and modest attenuation of the disease phenotype [12]. However, the HexB^−/−^;CCR2^−/−^ mice succumbed due to GM_2_ storage and neurodegeneration [12]. Therefore, this data implies that GM_2_ neuronal storage is sufficient to elicit neurodegeneration. The goal of this investigation was to evaluate whether GM_2_ ganglioside storage is necessary for neurodegeneration and neuroinflammation by pursuing β-hexosaminidase rescue experiments selectively in the neurons of HexB^−/−^ mice.

## Materials and methods

### Cloning

The Thy1-HEXB transgene was engineered by digesting the pTSC21k (9,098 bp) and pHEXB-IRES-HEXA [5] vectors with *Xho*I. Subsequently, HEXB was cloned into the pTSC21k vector containing the Thy1 promoter, which was donated by Dr Van der Putten [13]. The final clone was digested with *Eco*RV and *Bam*HI to check for orientation. The final construct was digested with *Not*I, and the 11.1 Kb fragment was send to the transgenic facility to generate transgenic Thy1-HexB mice.

### Animals and behavioral analyses

The use of mice and all manipulations and methods pertinent to this study were reviewed and approved by the University of Rochester Animal Resources committee (IACUC) as well as the Institutional Biosafety Committee and assigned protocol number 2003–142. HexB^+/−^ breeders were donated to us by Dr RL Proia (NIH/NIDDK; Bethesda MD, USA) on a 129 S background, and genotyped as previously described [7]. Thy1-HEXB transgenic mice were generated on 129 S background in the University of Rochester transgenic facility by microinjection of the approximately 11.1 Kb linearized DNA fragment into fertilized oocytes following standard procedures. Two founder mouse lines (F0) were developed, of which line no. 2 was able to transmit a functional copy of the Thy1-HEXB transgene to its offspring. F3 generation transgenic mice were then crossed into the HexB^+/−^ mouse and their offspring backcrossed into HexB^+/−^ mice to develop littermates of the desired genotype. Genotyping for the Thy1-HEXB transgene was facilitated by the following primer sets: 5′-AGTCCTGCCAGAATTTGATACC-3′ and 5′-ATTCCAAGTTCGACCATCC-3′ at Ta = 58°C. Genotyping for the HexB locus was performed as previously described [7].

Starting at 9 weeks of age, the mice were trained for 2 weeks on the rotarod appliance (Columbus Instruments, Columbus, OH, USA). At the age of 11 weeks, we began evaluating the behavioral performance of the mice by rotarod. Differences between the various animal groups were assessed by analysis of variance and *post hoc* analysis using the Tukey method.

#### *RNA isolation and cDNA synthesis*

Tissue was dissected out, frozen in isopentane chilled with dry ice, and stored in sterile tubes at −80°C until ready for RNA isolation. RNA was isolated using Trizol reagent (Invitrogen, Carlsbad, CA, USA), precipitated, and the concentration determined by spectrophotometry. A total of 2 μg of RNA was DNase-treated (Invitrogen) according to the manufacturer’s instructions. First-strand DNA was synthesized by using 2 μg of DNase-treated RNA, random hexamers, and Superscript II (Invitrogen) according to the manufacturer’s instructions.

#### *Quantification of mRNA abundance by real-time reverse transcription polymerase chain reaction (RT-PCR)*

Quantification of mRNA levels was accomplished using an iCycler (Bio-Rad, Hercules, CA, USA) and real time quantitative (q)RT-PCR with Taqman probes constructed with FAM as the fluorescent marker and Blackhole I quencher (Biosearch Technologies, Novato, CA, USA). Prior to PCR of the cDNA samples, PCR conditions were optimized for each mRNA to be analyzed. Standard curve reactions were performed by varying annealing temperatures, primer concentrations, and Taqman probe concentration. Serial dilution of the starting cDNA template demonstrated linear amplification over at least five orders of magnitude.

PCR reactions were performed in a volume of 25 μl and contained iQ Supermix (Bio-Rad; 0.625 U Taq, 0.8 mM dNTP, 3 mM Mg^2+^, 0.2 to 0.6 μM concentrations of each primer, 10 to 100 nM probe and 1 μl of cDNA sample). To ensure consistency, a master mix was first prepared containing all reagents except the cDNA sample. Primers were designed using the Primer Express (Applied Biosystems) and Oligo 6.83 programs (Molecular Biology Insights, Inc., Cascade, CO, USA). In general, PCR reaction conditions were the following: denaturation at 95°C for 3 minutes, followed by 40 cycles of amplification by denaturing at 95°C for 30 s, annealing at 60°C for 30 s and extension at 72°C for 60 s. For each real time PCR, a standard curve was performed to insure direct linear correlation between product yield (expressed as the number of cycles to reach threshold) and the amount of starting template. The correlation was always greater than r = 0.925. PCR reaction efficiency (e) was determined for each reaction. To correct for variations in starting RNA values, the level of ribosomal 18 S RNA or glyceraldehyde 3-phosphate dehydrogenase (GAPDH) RNA was determined for all samples and used to normalize all subsequent RNA determinations. Normalized threshold cycle (Ct) values were then transformed using the function expression = (1+ e) Ct, in order to determine the relative differences in transcript expression. Data were compared by analysis of variance (ANOVA) and Tukey’s post hoc tests, and by linear regression to determine correlations using the JMP statistics program (SAS Institute). A probability of *P* <0.05 was considered statistically significant. Cyclo-oxygenase 2 (COX-2) (Ta = 60°): UP-5′-tgacccccaaggctcaaata-3′, LP-5′-cccaggtcctcgcttatgatc-3′, PR-5′-ctttgcccagcacttcacccatcagtt-3′; GAPDH (Ta = 60°): UP-5-′cccaatgtgtccgtcgtg-3′, LP-5′-cctgcttcaccaccttcttg-3′, PR-5′-tgtcatatacttggcaggtttctccagg-3′; interleukin (IL)-1β (Ta = 60°): UP-5′-tcgctcagggtcacaagaaa-3′, LP-5′-atcagaggcaaggaggaaacac-3′; PR-5′-catggcacattctgttcaaagagagcctg-3′; IL-6 (Ta = 60o): UP-5′-ccagaaaccgctatgaagttcct-3′; LP-5′-caccagcatcagtcccaaga-3′; PR-5′-tctgcaagagacttccatccagttgcc-3′; tumor necrosis factor α (TNFα) (Ta = 55°): UP-5′-gacaaggctgccccgacta-3′; LP-5′-tttctcctggtatgagatagcaaatc-3′; PR-5′-ctcctcacccacaccgtcagcc-3′.

### Histology

The antibody used to detect CD45^+^ in our experiment was a rat anti-CD45^+^ monoclonal antibody commercially available from Serotec (Raleigh, NC, USA; cat. no. MCA43G) at 1:400 dilution, coupled with a rabbit anti-rat IgG secondary antibody (Jackson Immunoresearch; West Grove, PA, USA). Activated astrocytes were identified by a mouse anti-glial fibrillary acidic protein (GFAP) monoclonal antibody (1:400 dilution; Chemicon INTL, Temecula, CA, USA; clone GA-5). Activated denditric cells/microglia/macrophages were stained with a rat anti-major histocompatibility complex class II (MHC-II; Bachem, Torrance, CA, USA; clone ER-TR3). GM2 ganglioside was immunolocalized employing a mouse anti-*N*-acetyl GM2 monoclonal IgM antibody (Seikagaku, East Falmouth, MA, USA; clone MK1-16). These antibodies were coupled with appropriate secondary antibodies: rabbit anti-goat IgG biotin-conjugated, goat anti-mouse IgG Fab biotin conjugated, goat anti-rat IgG biotin-conjugated antibodies and goat anti-mouse IgM biotin conjugated, respectively (Jackson Immunoresearch). Human HEXB was detected by a goat anti-HEXB polyclonal antibody (Proia RL, NIH/NIDDK, Bethesda MD, U.S.A.) coupled with a rabbit anti-goat IgG secondary antibody (Jackson Immunoresearch). Visualization was performed utilizing 3,3-diaminobenzidine (DAB)-nickel as chromagen. The glass slides were then dehydrated through multiple ethanol solutions, cleared through xylene and coverslipped using DPX permanent mounting medium (Fluka, Neu-Ulm, Switzerland). The tissue sections were then studied under a BX51 Olympus light microscope and color microphotographic images were captured as described above. The total numbers of GFAP^+^ and MHC-II^+^ cells were counted in ten random microscopic fields (40 ×): in each field, the number of positive cells was counted, and averages and standard errors of mean were calculated for each area of the brain.

## Results

### Conditional restoration of β-hexosaminidase in neurons rescued HexB^−/−^ mice from GM_2_ ganglioside storage

**Figure 1:**
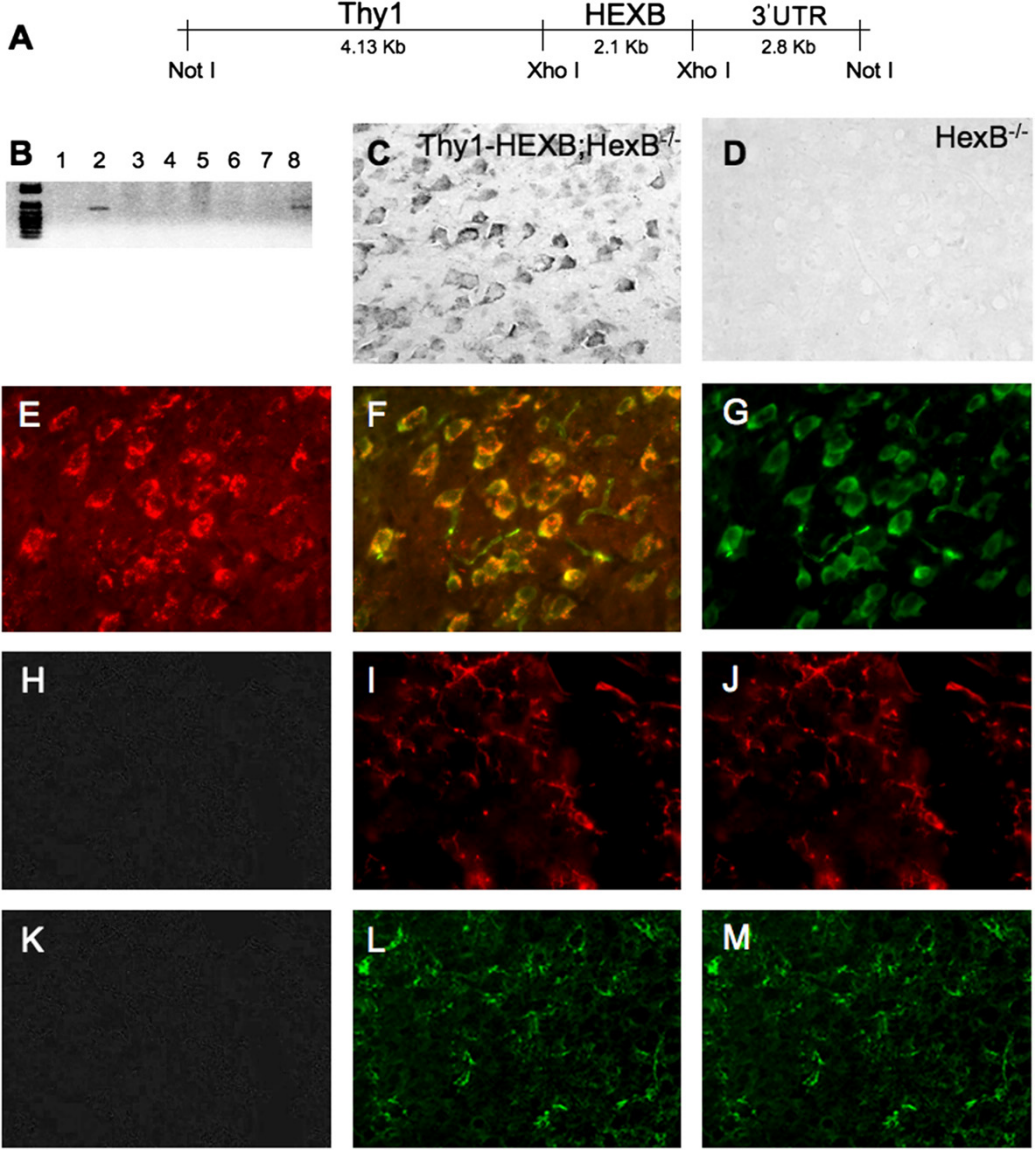
**Neuronal β-hexosaminidase (HEX) restitution in Sandhoff mice.** (**A**) The expression of human HEX β/β homodimer (HEXB) open reading frame was targeted to neurons by the Thy1 promoter. (**B**) Two transgenic mouse lines were generated. (**C**) β-Hexosaminidase expression was detected by immunohistochemistry employing an antibody raised specifically against human HEXB in histology sections harvested from Thy1-HEXB;HexB^−/−^ brains. (**D**) As expected, HexB^−/−^ littermates did not express any human HEXB protein. (**E**) The expression of HEXB (red) was confirmed by (**F**) double immunofluorescence (yellow overlap) in (**G**) neurons (green) in the brains of Thy1-HEXB;HexB^−/−^ mice. (**H**) The lack of HEXB expression was confirmed (**I**) by double immunofluorescence in (**J**) MHC-II^+^ microglia/macrophages. Similarly, there (**K**) was lack of HEXB expression in astrocytes as detected (**L**) by the lack of double immunofluorescence in (**M**) glial fibrillary acidic protein (GFAP)^+^ astrocytes.

Conditional restitution of β-hexosaminidase in the neurons of HexB^−/−^ mice was accomplished by driving the expression of the human HEXB cDNA by the neuron specific Thy1 promoter in transgenic mice (Figure 1A). Two founder lines were generated in the 129 S strain background, of which line no. 2 successfully transmitted the transgene to the offspring (Figure 1B). Transgenic mice derived from founder mouse line no. 2 were subsequently crossed into the HexB^−/−^ knockout mouse colony (129 S background strain) for the development of Thy1-HEXB;HexB^−/−^ compound mice. The Thy1-HEXB;HexB^−/−^ were characterized by expression of human HEXB in neurons (Figure 1C). As expected, HexB^−/−^ mice did not display any human HEXB expression (Figure 1D). Double immunofluorescence analysis confirmed the expression of human HEXB in neuron (Figure 1E-G), but not MHC-II^+^ microglia/macrophages (Figure 1H-J) or GFAP^+^ astrocytes (Figure 1K-M).

**Figure 2:**
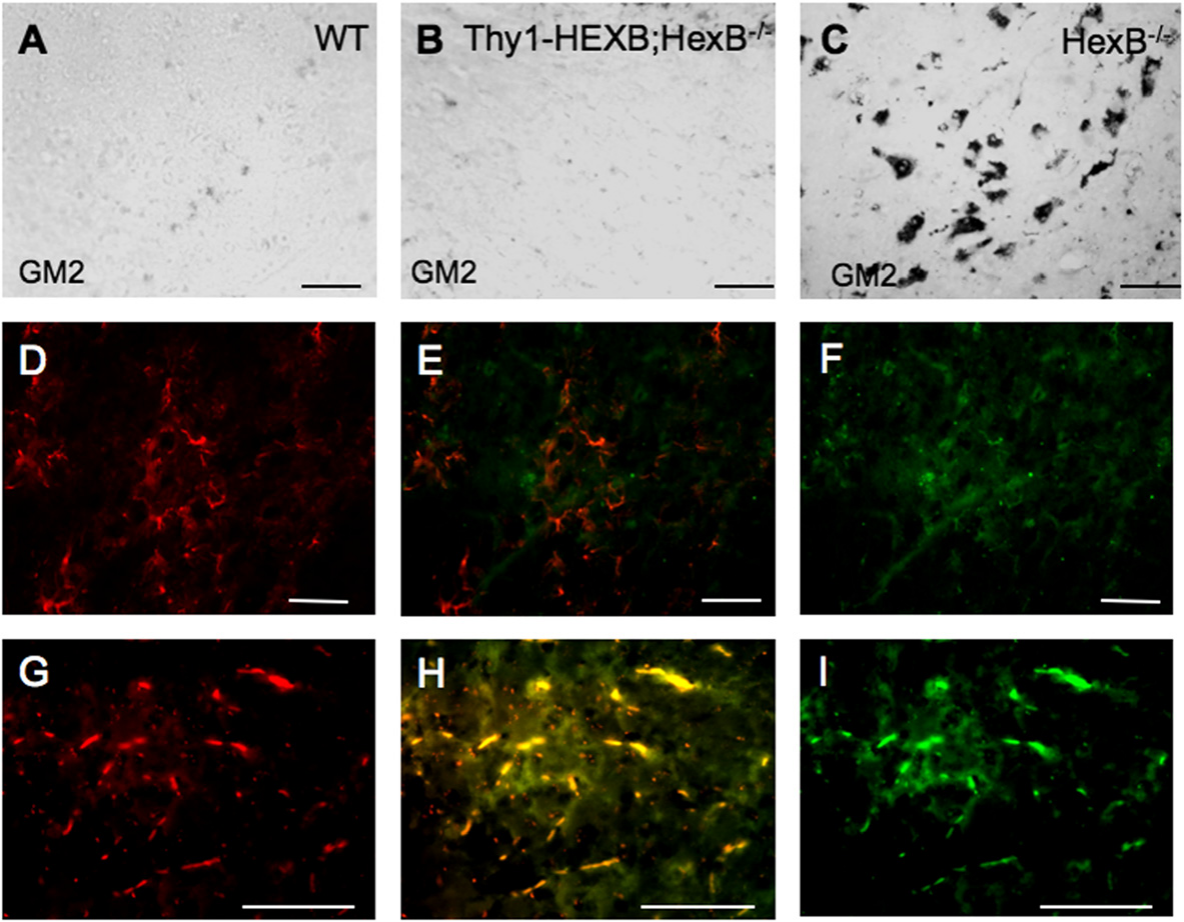
**GM**_**2**_**ganglioside storage in the Thy1-HEXB;HexB**^**−/−**^**brain.** (**A**) Wild-type mice did not display any GM_2_ storage as detected by immunohistochemistry (black staining) employing a monoclonal antibody. (**B**) The expression of human HEXB in the neurons of HexB^−/−^ mice driven by the Thy1 promoter resulted amelioration of GM_2_ storage, as seen by the lack of any significant GM_2_ immunoreactivity in 4-month-old Thy1-HEXB;HexB^−/−^ mouse brain compared to (**C**) HexB^−/−^ littermates. (**D**) Glial fibrillary acidic protein (GFAP)^+^ astrocytes (**E**) did not contain, as detected by the lack of double immunofluorescence, the presence of (**F**) GM_2_ ganglioside. However, (**G**) major histocompatibility complex class II (MHC-II)^+^ microglia/macrophages contained, as detected (**H**) by double immunofluorescence, the presence of (**I**) GM_2_ ganglioside. Bar = 100 μM. HEXB = β-hexosaminidase β/β homodimer.

Immunohistochemical analysis revealed the lack of GM2 ganglioside in wild-type mice (Figure 2A) and comparable low levels in the Thy1-HEXB;HexB^−/−^ compound mice (Figure 2B), compared to HexB^−/−^ knockout mice, which are known to develop GM2 storage in neurons (Figure 2C). Further analysis by double immunofluorescence analysis revealed that GFAP^+^ astrocytes do not contain any GM2 ganglioside in the Thy1-HEXB;HexB^−/−^ mouse model (Figure 2D-F). Conversely, MHC-II^+^ microglia/macrophages displayed the presence of GM_2_ ganglioside content in the Thy1-HEXB;HexB^−/−^ mouse model despite the restoration of HEXB activity in neurons (Figure 2G-I).

### β-Hexosaminidase restitution in the brain of HexB^−/−^ mice did not abolish brain inflammation

**Figure 3:**
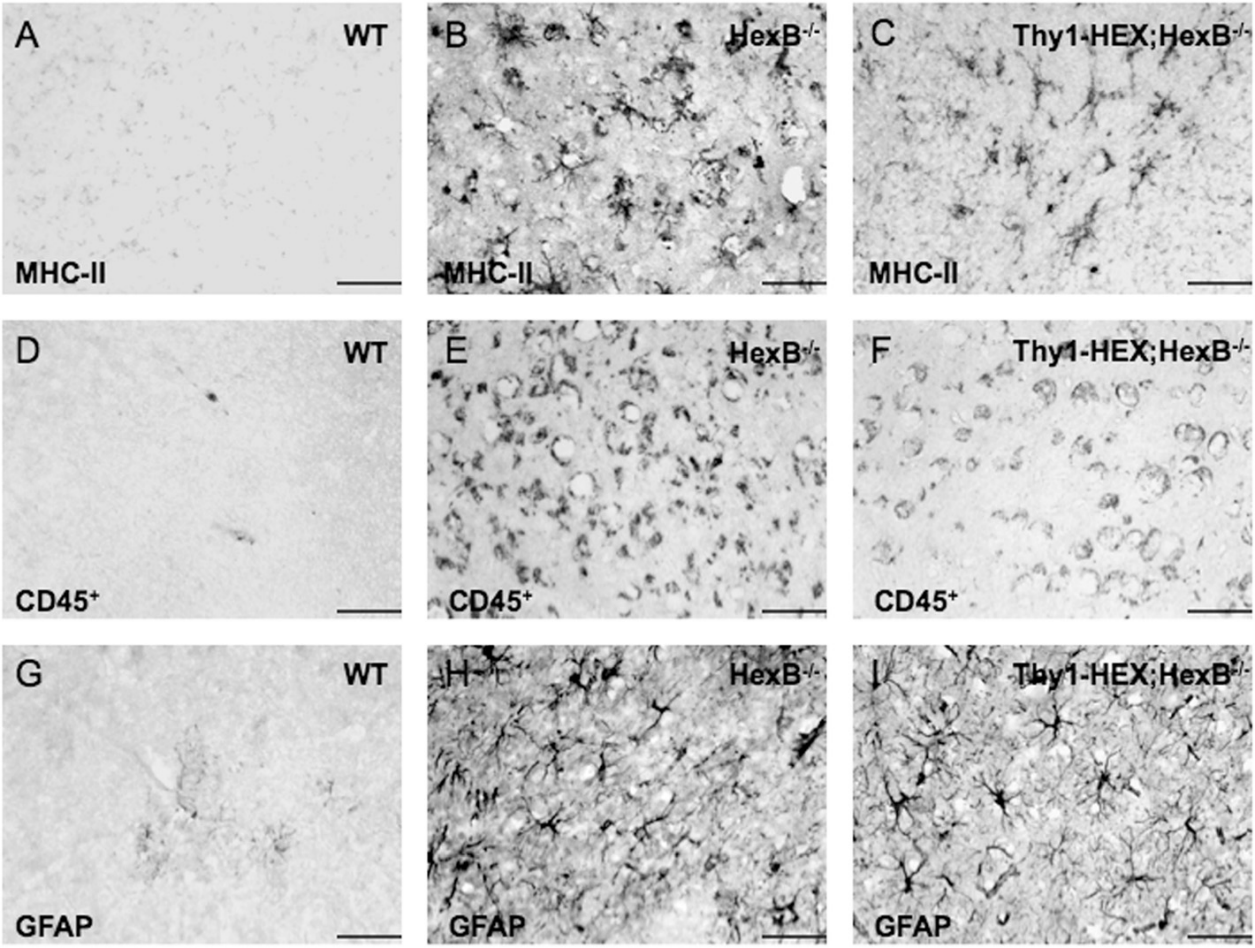
**Neuronal β-hexosaminidase (HEX) restitution does not reduce brain inflammation in adult HexB**^**−/−**^**mice.** (**A**) Adult wild-type brains lacked any major histocompatibility complex class II (MHC-II) immunoreactive cells, whereas (**B**) HexB^−/−^ brains were characterized by the presence of MHC-II positive cells, a marker of myeloid-derived immune cells, throughout the brain parenchyma. (**C**) The 4-month-old Thy1-HEXB;HexB^−/−^ littermates were also characterized by the presence of MHC-II positive cells in their brain. Another marker of myeloid-derived immune cells expressed in high levels by peripheral blood macrophages/monocytes intensely is CD45^+^. (**D**) Wild-type mice were characterized by the lack of CD45^+^-positive cells, whereas (**E**) HexB^−/−^ mice display high numbers of CD45^+^ cells, also (**F**) present in the brains of Thy1-HEXB;HexB^−/−^ littermates. Moreover, we evaluated the presence of reactive astrocytes in the mouse brains by glial fibrillary acidic protein (GFAP) immunohistochemistry. (**G**) Wild-type mice lacked GFAP^+^ cells (activated astrocytes) in their cortex. Conversely, (**H**) HexB^−/−^ mice (4 months old) displayed GFAP^+^ astrocytes throughout their brain parenchyma, as well as **(I)** in the brain of Thy1-HEXB;HexB^−/−^ littermates. Bar = 100 μM.

**Figure 4:**
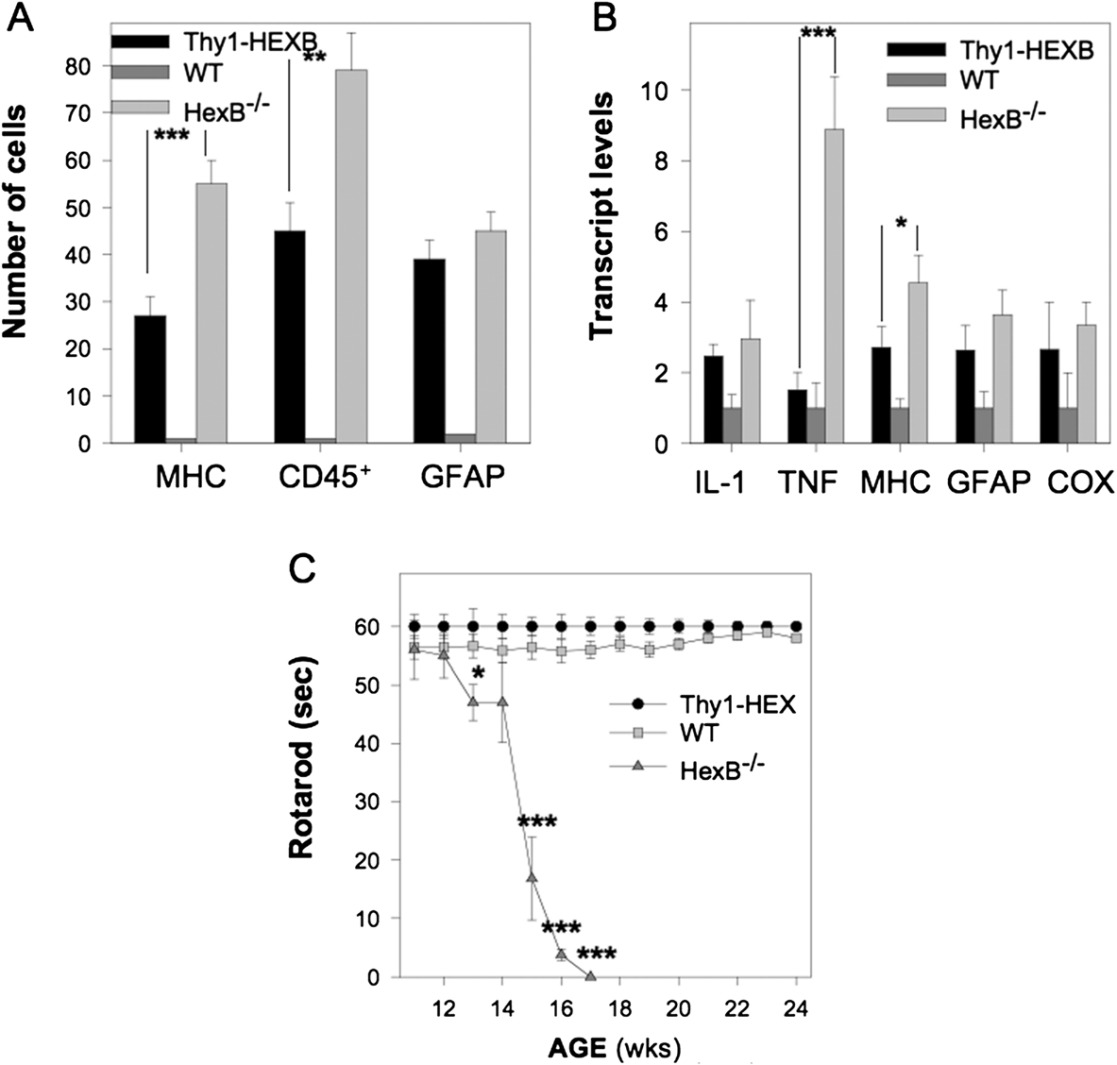
**Rescue of HexB**^**−/−**^**mice from GM**_**2**_**storage has limited effect on neuroinflammation.** Targeted rescue of β-hexosaminidase deficiency selectively in the neurons of HexB^−/−^ mice (**A**) significantly reduced the number of major histocompatibility complex class II (MHC-II)^**+**^ and CD45^**+**^ cells in the brain, but not of glial fibrillary acidic protein (GFAP)^**+**^ astrocytes. (**B**) We also observed downregulation in the expression of the tumor necrosis factor α (TNFα) and MHC-II genes, but not of interleukin (IL)-1β, GFAP or cyclo-oxygenase 2 (COX-2) in 4-month-old Thy1-HexB;HexB^−/−^ mice compared to HexB^−/−^ littermates. (**C**) Interestingly, the Thy1-HexB;HexB^−/−^ mice were characterized by fully rescued neurological phenotype, assessed by rotarod, even in the presence of reactive astrocytes and retained brain inflammation. Caspase-3 levels were evaluated in the brain as a measure of neurodegeneration. ***P <0.001; **P <0.01; *P <0.05.

Brain inflammation was assessed by evaluating transcript (mRNA) levels for key inflammatory genes in total RNA brain extracts, as well as by assessing the presence of reactive astrocytes and microglia in histology brain sections. Astrocyte activation was evaluated by GFAP immunohistochemistry. Microglia activation was assessed by MHC-II^+^ and CD45^+^ immunohistochemistry. As anticipated, HexB^−/−^ mice were characterized by the presence of GFAP, MHC-II^+^ and CD45^+^ cells throughout their brain parenchyma (Figures 3B) and (4A), as well as significant upregulation in key inflammatory genes in their brain (Figure 4B). Conversely, we observed a reduction in the number of MHC-II^+^ and CD45^+^ cells in the brain parenchyma of Thy1-HEXB; HexB^−/−^ mice at a significant degree (Figure 4A). CD45^+^ immunoreactivity was employed as a marker of infiltrating macrophages/monocytes, as CD45^+^ is expressed at high levels by activated myeloid derived immune cells [7,12]. CD45^+^ is also expressed by other immune cells, however at low levels (detectable by flow cytometry) that fall below the detection threshold of our immunohistochemical method. Moreover, there was a significant downregulation of TNFα and MHC-II transcript in Thy1-HEXB;HexB^−/−^ brains (Figure 4B). Interestingly, there were no changes in the number of activated astrocytes in the HexB^−/−^ versus Thy1-HEXB;HexB^−/−^ mouse brains (Figures 3H-I and 4A). To this end, there was a significant downregulation in some inflammatory genes (Figure 4B), including TNFα and MHC-II, but not in others (GFAP, IL-1β, COX-2).

### Restoration of neuronal function rescues HexB^−/−^ mice from neurodegeneration

**Figure 5:**
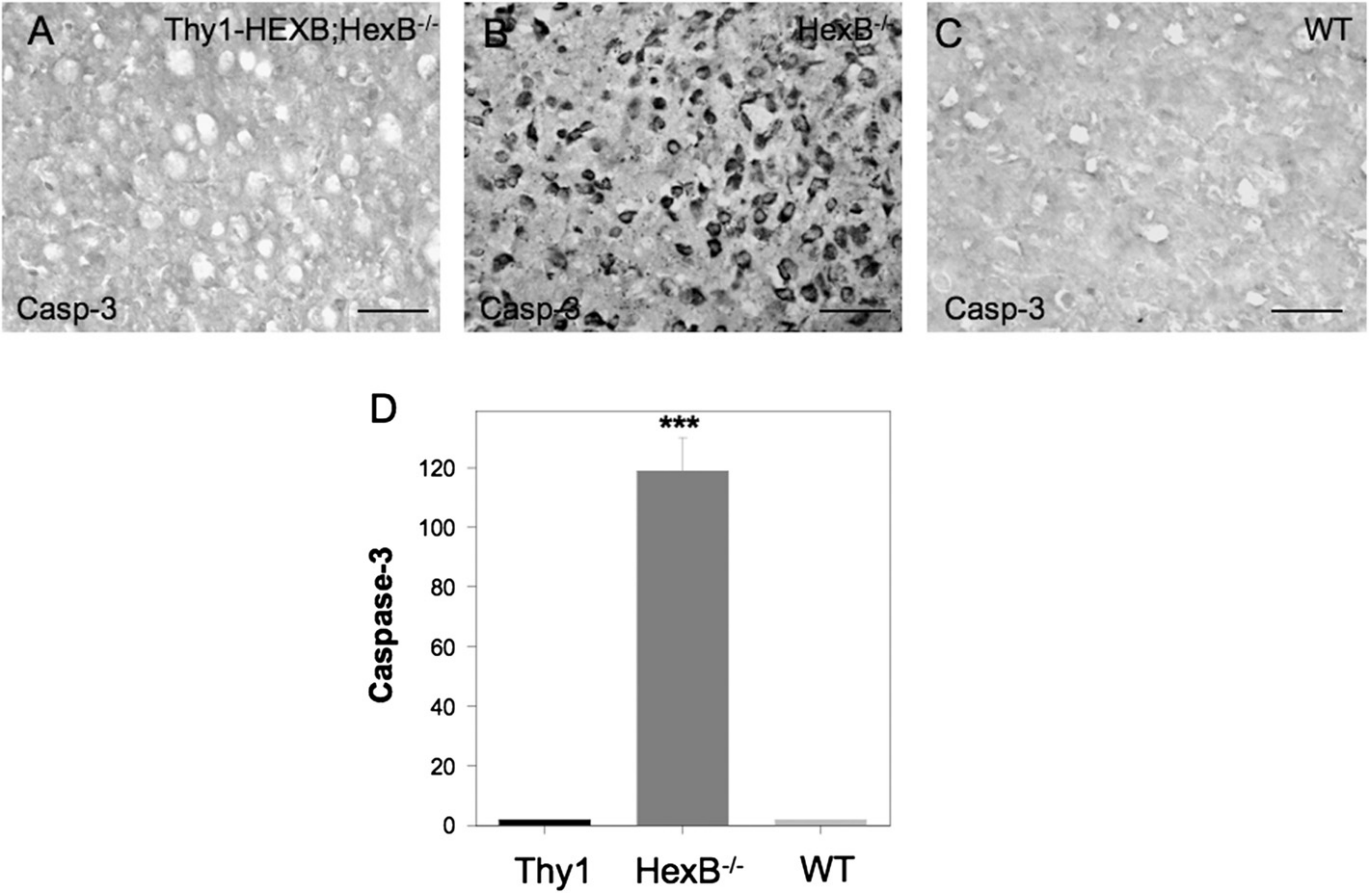
**Caspase-3 expression is reduced in Thy1-HexB;HexB**^**−/−**^**mice.** Caspase-3 immunoreactivity was employed as a measure of neurodegeneration. (**A**) Thy1-HexB;HexB^−/−^ mice lacked any detectable caspase-3 expression, as assessed by immunohistochemistry. **(B)** Conversely, HexB^−/−^ mice displayed intense caspase-3 immunoreactivity located in cortical neurons. (**C**) Wild-type mice did not present any caspase-3 immunostaining in their brain. (**D**) Cell counts of caspase-3 immunopositive cells was reduced significantly in Thy1-HexB;HexB^−/−^ mice, to levels observed in wild-type mice. Bar = 100 μM. ***P <0.001; **P <0.01; *P <0.05.

Neuronal β-hexosaminidase restitution in the Thy1-HEXB;HexB^−/−^ mice led to amelioration of neurodegeneration and normalization of the clinical phenotype, despite the partial reduction in neuroinflammation. Neurodegeneration was evaluated clinically by rotarod test as well as by caspase-3 immunohistochemistry in brain histology sections. As expected, HexB^−/−^ mice were characterized by a significant decline in motor behavior starting at 14 weeks of age, followed by complete clinical deterioration by 17 weeks of age (Figure 4C) when they were killed according to approved animal use protocols. Conversely, Thy1-HEXB;HexB^−/−^ mice performed on the rotarod test similar to wild-type mice for the total duration of the experiment (Figure 4C). Further, we have followed these mice for over 12 months and they continuously display normal motor behavior. Furthermore, caspase-3 immunoreactivity was reduced to wild-type levels in the Thy1-HEXB;HexB^−/−^ mice, whereas HexB^−/−^ mice displayed extensive caspase-3 immunostaining (Figure 5).

## Discussion

In order to elucidate the role of GM_2_ ganglioside storage in neurodegeneration and neuroinflammation, we restored β-hexosaminidase selectively in the neurons HexB^−/−^ mice targeting the expression of the human HEXB by the Thy1 neuron specific promoter selectively in neurons. Our results demonstrated that Thy1-HEXB;HexB^−/−^ mice were rescued from GM_2_ neuronal storage and displayed normal clinical phenotype. This set of data proves that β-hexosaminidase restoration in neurons is sufficient for the rescue of mice from GM_2_ gangliosidosis. Although this finding was anticipated, as β-hexosaminidase deficiency is pathognomonic in GM_2_ gangliosidosis [1], we observed a significant number of prevailing MHC-II^+^ cells, a marker of activated microglia and myeloid derived immune cells, including peripheral blood macrophages and monocytes, in the brain of the Thy1-HEXB;HexB^−/−^ mice. Upon further analysis, we identified the presence of GM_2_ ganglioside specifically in MHC-II^+^ cells (Figure 2); conversely, we did not find GM_2_ in astrocytes. Moreover, we confirmed the identity of cells expressing human HEXB in the Thy1-HEXB;HexB^−/−^ mice to be selectively neurons. Taken together, these data suggest that GM_2_ is likely phagocytosed by microglia/macrophages, underlying the increased number of MHC-II^+^ cells in the brain of the Thy1-HEXB;HexB^−/−^ mice (Figure 4). The presence of GM_2_ in microglia/macrophages is surprising, as it was anticipated that human HEXB would be secreted by neurons in the extracellular milieu and from there taken up by neighboring cells, a key characteristic of lysosomal enzymes [14]. The presence of GM_2_ along with lack of human HEXB in microglia/macrophages suggests that HEXB crosscorrection between different cell types in the Thy1-HEXB;HexB^−/−^ model is not efficient. The number of GFAP^+^ astrocytes was also increased in the HexB^−/−^ and Thy1-HEXB;HexB^−/−^ mice compared to littermate controls. Moreover, we did not observe the colocalization of GFAP with HEXB or GM2, implying that astrocyte activation is presumably secondary to microglia/macrophage activation. Taken together, our data suggest that in the Thy1-HEXB;HexB^−/−^ mouse model, HEXB is expressed selectively in neurons under the Thy1 promoter, leading to GM_2_ clearance in neurons. Microglia and infiltrating macrophages also participate in GM_2_ clearance, but unlike neurons, they are unable to catabolize GM_2_, which accumulates over time in their cytoplasm and activates them. Astrocytes do not express HEXB nor phagocytose GM_2_, but appear to become activated presumably from cell-cell interactions (for example microglia-astrocyte). Previous studies in our laboratory have also associated TNFα upregulation with microglia activation and monocyte/macrophage infiltration in the HexB^−/−^ brain [12]. To this end, we have previously demonstrated how activated microglia are capable of activating in turn astrocytes via IL-1β and TNFα [15], cytokines which were found elevated in the Thy1-HEXB;HexB^−/−^ mouse brains. Despite the presence of neuroinflammation, Thy1-HEXB;HexB^−/−^ mice exhibit no signs of neurodegeneration, both clinical and neuropathological (expression of the proapoptotic gene caspase-3 was ameliorated). Our results suggest that the attendant neuroinflammation is not sufficient to elicit neurodegeneration or to affect normal mouse behavior. This finding agrees with previous report by other investigators that have shown neuroinflammation to play a contributory, but not pathognomonic, role in GM_2_ gangliosidosis [4,5,8] and other neurodegenerative diseases [10,11,16].

In conclusion, the present study demonstrated that restoration of neuronal function is paramount for the treatment of neurodegeneration in the Sandhoff disease mouse model, notwithstanding the contributory role of neuroinflammation to neurodegeneration. Moreover, our study demonstrated that the level of attendant neuroinflammation in the Thy1-HEXB;HexB^−/−^ model is insufficient to elicit neurodegenerative or behavioral changes in mice.

## Competing interests

The authors state that there are no conflicts, financial or other, in regards to the generation of this manuscript.

## Authors’ contributions

SK: organized research program, analyzed data and wrote paper. SMB: contributed to data generation. RHT: contributed to data generation. JHM: contributed to data generation. JAO: contributed to data generation. MKO’B: contributed to the composition of the manuscript and data analysis. All authors read and approved the final manuscript.
